# Prevalence and outcome of invasive pulmonary aspergillosis in critically ill patients with liver cirrhosis: an observational study

**DOI:** 10.1038/s41598-019-48183-4

**Published:** 2019-08-15

**Authors:** Tobias Lahmer, Andreas Brandl, Sebastian Rasch, Gonzalo Batres Baires, Roland M. Schmid, Wolfgang Huber, Ulrich Mayr

**Affiliations:** 1 Klinik und Poliklinik für Innere Medizin II, Klinikum rechts der Isar der Technischen Universität München, Munich, Germany; 2 Department of General, Visceral and Transplantation Surgery and Department of General, Visceral, Vascular and Thoracic Surgery, Campus Virchow and Mitte, Charité, Universitätsmedizin Berlin, Berlin, Germany

**Keywords:** Risk factors, Diagnostic markers

## Abstract

Invasive pulmonary aspergillosis (IPA) is an important cause of morbidity/mortality in critically ill patients with endstage liver disease. Therefore, aim of this study is to predict the prevalence and outcome of IPA in critically ill patients with underlying liver cirrhosis and evaluation of the necessity Glactomannan (GM) screening in serum and bronchoalveolar lavage (BAL) in this cohort. In total 12 out of 84 patients (14%) had probable IPA. The mean optical density index (ODI) bronchoalveolar lavage (BAL) GM index was 3.6 ± 1.5 (Range: 1.7–5.7). An overall sensitivity of 90% (95% CI 86–96%) and specificity of 85% (95% CI 81–88%) was found for the BAL GM in IPA. Acute Physiology And Chronic Health Evaluation (APACHE II), sequential organ failure assessment (SOFA) as well the model of endstage liver disease (MELD) score were significantly higher in the probable IPA group as compared to the No IPA group (26 versus 21, p < 0.001 and 14 versus 10, p < 0.044). Length of intensive care unit (ICU) stay was significantly longer in probable IPA patients (16 versus 10 days, p < 0.027) and mortality rate was significantly higher in probable IPA patients (100% versus 65%, p < 0.001) as compared to No IPA patients. APACHE II and MELD score were independently associated with higher mortality rate using multivariate logistic regression (p = 0.025 and p = 0.034). In conclusion, IPA has a relevant impact on outcome. Screening for IPA is indicated, easy to perform and a necessity to improve outcome.

## Introduction

Invasive pulmonary aspergillosis (IPA) is commonly known as a severe opportunistic infection in immunocompromised patients^1^. Recently, IPA has also been increasingly recognized in non –neutropenic critically ill patients as an emerging disease^2^.

Till today mostly described in small studies and case series, end stage liver cirrhosis is poorly recognized as a relevant underlying disease for IPA^3,4^.

It ist well known that invasive fungal infections lead to immunodeficiency based not only on various genetic influences but it also affects the innate and adaptive immune systems^5^.

In our study population the reasons for the development of IPA are diverse. Not only underlying host factors, but also immunoregulatory abnormalities following critical illness and/or liver cirrhosis can induce a state of immunoparalysis^2,6^.

The high incidence of infections may be explained by the so called cirrhosis associated immune dysfunction (CAID) which combines immunodeficiency and disturbance of specific immune system cells, including not only neutrophils and monocytes but also T and B cells^6,7^.

This leads into a multifactorial process of continously overstimulation of immune cells, hypersplenism and splenic pooling of immune system called “immune paralysis”^6,8^.

This “ immune paralysis” results in an inadequate host response to potential infections, especially fungal diseases and may be supported by acute conditions e.g. severe sepsis or acute respiratory distress syndrome (ARDS) and may explain the devasting high morbidity and mortality among critically ill patients with end stage liver disease^9,10^.

Moreover, one major problem in non-neutropenic patients is the timely and correct diagnosis of IPA which is in most cases combined with non specific clinical presentations and limited accuracey of diagnostic tests as compared to neutropenic patients^2^.

Patients with liver cirrhosis are at risk for IPA. Therefore, aim of this study is to assess the prevalence and outcome of IPA in critically ill patients with underlying liver cirrhosis and evaluating the necessity of Glactomannan (GM) screening in this cohort.

## Results

A total of 84 critically ill patients with underlying liver cirrhosis were included in the study.

The demographic and clinical characteristics of the patients are presented in Table 1.

**Table 1:** presents demographic and clinical characteristics of the patients and risk factors for invasive pulmonary aspergillosis.

Patient characteristics	Overall study cohort	Probable IPA	No IPA	p-valvue
Number of patients (n)	84	12 (14)	72 (86)	
Male (n, %)	53 (65)	9 (75)	44 (61)	p = 0.203
Age (years)	59 ± 11	59 ± 8	60 ± 12	p = 0.910
***Etiology of cirrhosis*** (***n***, ***%***):				
Alcoholic liver disease	59 (71)	9 (75)	50 (70)	p = 0.314
Chronic viral hepatitis B/C	8 (9)	0 (0)	8 (9)	**p** **<** **0**.**001**
Autoimmune	3 (4)	1 (8)	2 (3)	p = 0.288
Others or unknown	14 (16)	2 (17)	12 (18)	p = 0.344
CHILD-Pugh A	0	0		
CHILD-Pugh B	13 (16)	0	13 (18)	**p** **<** **0**.**001**
CHILD-Pugh C	71 (84)	12 (100)	59 (82)	p = 0.098
Decompensated cirrhosis	84 (100)	12 (100)	72 (100)	p = 0.452
ACLF Score:				
2 (n)	34	0	4	p = 0.065
3 (n)	50	12	38	p = 0.089
***Clinical findings*** (***n***, ***%***):				
Fever	47 (56)	8 (66)	39 (54)	p = 0.104
Cough	32 (38)	5 (42)	27 (38)	p = 0.214
Hemoptysis	5 (6)	1 (8)	4 (6)	p = 0.188
***Baseline characteristics***:				
APACHE II score	21 ± 7	26 ± 8	21 ± 7	**p** **<** **0**.**001**
SOFA score	11 ± 3	14 ± 4	10 ± 4	**p** **=** **0**.**044**
MELD score	26 ± 8	31 ± 8	24 ± 8	**p** **=** **0**.**034**
Leukocytes G/l	11, 5 ± 6, 5	12, 5 ± 5, 3	11, 3 ± 6, 8	p = 0.087
C-reactive protein mg/dl	5, 5 ± 4, 5	7, 8 ± 5, 5	5 ± 3, 5	p = 0.287
Procalcitonin ng/ml	2, 5 ± 1, 3	6, 7 ± 1, 7	2, 7 ± 1, 1	p = 0.113
***Reason for ICU admission*** (***n***, ***%***):				
Pneumonia	23 (27)	4 (33)	19 (26)	p = 0.108
Sepsis	28 (33)	5 (42)	23 (32)	p = 0.098
HRS	19 (23)	2 (17)	17 (24)	p = 0.122
Gastrointestinal bleeding	14 (17)	1 (8)	13 (18)	p = 0.088
***Risk factors for IPA*** (***n***, ***%***):				
Neutropenia	0	0	0	p = 0.450
Diabetes mellitus	3 (4)	1 (8)	2 (3)	p = 0.097
Hepatic carcinoma	3 (4)	0	3 (4)	p = 0.054
Chronic lung disease	4 (5)	1 (8)	3 (4)	p = 0.132
Mechanical ventilation(n, %)	80 (95)	12 (100)	68 (95)	p = 0.145
Hemodialysis	46 (55)	11 (90)	35 (48)	**p** **=** **0**.**045**
Glucocorticoid therapy	11 (13)	2 (16)	9 (13)	p = 0.110
***Broad-spectrum antibiotics usage***				
One antibiotic	13 (15)	0	13 (18)	**p** **=** **0**.**032**
Two antibiotics	71 (85)	12 (100)	59 (82)	p = 0.088

Invasive pulmonary aspergillosis (IPA), Acute Physiology And Chronic Health Evaluation (APACHE), Sequential Organ Failure Assessment score (SOFA), model of endstage liver disease (MELD). Data are presented as mean ± standard deviation, statistical significant parameters are highlighted. **ACLF Score** (**acute on chronic liver failure**).

Twelve cases of probable IPA (14%) were observed. IPA could be excluded in the remaining 72 patients.

### Basic characteristics

Divided in patients with IPA and No IPA significant differences could be observed:

APACHE II, SOFA as well the MELD (model of endstage liver disease) score were significantly higher in the probable IPA group as compared to the No IPA group (26 versus 21, p < 0.001 and 14 versus 10, p < 0.044). The acute on chronic liver failure (ACLF) score was not statistical significant in both groups, however, ACLF grade 3 was only found in the IPA group, ACLF grade 2 and 3 in the No IPA group. Moreover, probable IPA patients needed significantly more renal replacement therapy (90% versus 48%, p < 0.045) and in the No-IPA group broad spectrum antibiotic were significantly less often often used as compared to the IPA group (p = 0.032).

No differencies could be observed in other risk factors for IPA: frequency of neutropenia (p = 0.450), diabetes mellitus (p = 0.097), hepatic carcinoma (p = 0.054), chronic lung disease (p = 0.132), mechanical ventilation (p = 0.145) and glucocorticoid therapy (p = 0.110). In case of suspected alcoholic steato hepatitis 40 mg of intravenous prednisolone were used in all patients with glucocorticoid therapy, mean treatment time was 7 ± 3 days. Other immunosuppressive medication was not used in this study population.

Main reasons for intensive care unit (ICU) admission were in both groups without statistical significance pneumonia (p = 0.108), sepsis (p = 0.098), hepato-renal-syndrome (p = 0.122) and gastrointestinal bleeding (p = 0.088).

Main causes of end stage liver disease were in both groups heavy alcohol intake (p = 0.314), followed by significantly more hepatitis B and C in the No-IPA group (p < 0.001), autoimmune hepatitis (p = 0.258) and unknown reasons (p = 0.344) (see Table 1). This is in the line with the CHILD Pugh classifications which were in both groups mainly CHILD C with significantly more CHILD B liver cirrhosis in the No-IPA group (p < 0.001) (see Table 1). All patients had decompensated liver cirrhosis (p = 0.452).

Leukocyte count, C-reactive protein and procalcitonin levels were not statistical significant different in patients with probable and No IPA (p = 0.087; p = 0.287; p = 0.113).

### Mycological characteristics

The mean intensive care unit (ICU) stay till diagnosis of IPA was 6 ± 4 days (see Table 2). Positive Galctomannan out of BAL (BAL-GM) fluid could be detected in 12 (14%) out of 84 patients. 5 ± 3 BAL-GM in the IPA and 2 ± 1 BAL-GM tests were performed in the No-IPA group. The mean ODI BAL GM index was 3.6 ± 1.5 (Range: 1.7–5.7). The BAL-GM ODI of the other patients were in repeated measurements <1 (see Table 2). Four patients without mechanical ventilation were screened by serum GM with no evidence for IPA with an overall ODI of <0.5.

**Table 2:** presents mycological parameters and treatment.

	Overall study cohort	Probable IPA	No IPA	p -value
Length of ICU stay (d)	11 ± 6	16 ± 7	10 ± 7	**p** **=** **0**.**027**
ICU stay till diagnosis (d)		6 ± 4		
***GM test per patient*** (***n***)				
Serum		0	4 ± 1	
BAL		5 ± 3	2 ± 1	
***Galactomannan*** (***ODI***; ***range***)				
Serum			<0, 5	
BAL		3.6 ± 1.5 (1.7–5.7)	<1	
***Glactomannan*** (***ODI***; ***range***) ***after therapy***:				
BAL		2.8 ± 1.2 (1.6–4.2)		
***Microbiological findings***:				
*Aspergillus spp*.		10 (83)		
***Galactomannan*** (***BAL***):				
- Sensitivity (%)		90% (95% CI 86–96%)		
- Specificity (%)		85% (95% CI 81–88%)		
***Laboratory Parameters on day of IPA diagnosis***:				
Leukocytes G/l		12.7 ± 4.4		p = 0.144
C-reactive protein mg/dl		5.8 ± 2.8		p = 0.224
Procalcitonin ng/ml		4.9 ± 2.3		p = 0.127
***Imaging findings*** (***n***, ***%***)				
Changes in bilateral lung fields	34 (40)	7 (58)	27 (38)	p = 0.112
Bilateral lung fields diffused	34 (40)	5 (42)	29 (40)	p = 0.164
Unilateral findings	16 (19)	0	16 (22)0	**p** **=** **0**.**034**
Halo or air-crescent sign	0	0	0	p = 0.335
***Treatment*** (***n***)				
Voriconazole		1		
liposomal Amphotericin B		11		
Mortality rate (%)		100	65	**p** **<** **0**.**001**

Invasive pulmonary aspergillosis (IPA), bronchoalveolar lavage (BAL), Galactomannan (GM), Data are presented as mean ± standard deviation, statistical significant parameters are highlighted.

An overall sensitivity of 90% (95% CI 86–96%) and specificity of 85% (95% CI 81–88%) was found for the BAL GM in IPA.

In detail a sensitivity of 98% (95% CI 95–100%) and specificity of 86% (95% CI 88–96%) for *probable* IPA and a sensitivity of 88% (95% CI 82–90%) and specificity of 82% (95% CI 78–84%) for *possible* IPA was found (see Table 2).

Radiological findings, especially IPA specific findings like Halo or air-crescent sign as well clinical findings revealed no-differences between the IPA and No-IPA group, with one exception of more unilateral findings in the ct scan in the No-IPA group (p = 0.034).

*Aspergillus fumigatus* could be identified by culture in ten (83%) out of twelve IPA cases. As all these cases presented highly positive BAL GM parameters, cultural growth was therefore interpreted as infection and not as colonization. Follow up examinations after initiated antimycotic therapy revealed in 2 patients ongoing growth of *Aspergillus spp*.

Cultures in No-IPA revealed only bacterial and in 21 cases *Candida spp*. findings. The *Candida spp*. findings were interpreted as colonization.

No difference of Leukocyte count, C-reactive protein and procalcitonin levels could be detected in the IPA group between baseline measurements and day of IPA diagnosis (p = 0.144; p = 0.224; p = 0.127).

Eleven patients out of twelve patients of the IPA group received for tretament liposomal amphotericin B and one patient voriconazole. Susceptibility testing was not routinely performed.

Follow up BAL GM after initiation of treatment revealed a decreased ODI of 2.8 ± 1.2 (range: 1.6–4.2) (see Table 2).

### Outcome characteristics

Length of ICU stay was significantly longer in probable IPA patients (16 versus 10 days, p < 0.027) and mortality rate was significantly higher in probable IPA patients (100% versus 65%, p < 0.001) as compared to No IPA patients.

Risk factors for death included higher APACHE II, SOFA and MELD score. APACHE II and MELD score were independently associated with higher mortality rates using multivariate logistic regression (p = 0.025 and P = 0.034) (see Table 2).

## Discussion

In our study a prevalence of 14% for IPA in critically ill patients with underlying endstage liver cirrhosis could be detected.

Comparable high prevalence rates for IPA of up to 16%, combined with a high mortality rate of nearly 100%, are till today only described for a subgroup of patients with liver disease, namley patients with acute severe alcoholic hepatitis^11,12^.

This might be explained by the fact that in those study cohorts systemic gluccocorticoid use is the mainstay of the therapy which is well recognized as a risk factor in the EORTC/MSG guidelines^13^.

However, as reported in our study, end stage liver cirrhosis was also without gluccocorticoid therapy a relevant host factor devleoping IPA, especially if they are critically ill.

As mentioned above the combination of CAID caused immunodeficiency with alterations of the adaptive and the innate immune system contribute to a high vulnerability for infections^6^.

Following the European Organization for Research and Treatment of Cancer/Invasive Fungal Infections Cooperative Group and the National Institute of Allergy and Infectious Diseases Mycoses Study Group Consensus Group EORTC/MSG guidelines host factors may be the major hint to diagnose IPA. However, typical risk factors mentioned in other studies e.g. neutropenia or diabetes mellitus for developing IPA were not statistical significant different in both groups in this study.

As reported in a recent study an elevated prevalence for IPA was not found in patients with end stage liver cirrhosis if they are not critically ill^14^.

Although, liver cirrhosis, foremost decompensated and CHILD C liver cirrhosis, were included in the adapted EORTC/MSG host factors for this study, it should be mentioned that till today not clearly defined and recognized the severtity of critical illness beyond end stage liver disaese itself reported in our study expressed in an high APACHE II, SOFA and high MELD score and the lenght of the ICU stay may also play a susbtantial role as a sgnificant host factor in developing IPA.

Another explanation beyond critical illness and/or liver cirrhosis induced immunoparalysis for the high prevalence of IPA in non neutropenic patients might be that invasive fungal infections in ICU patients are often missed or diagnosed late due to non-specific signs and symptoms and the difficulty of differentiating between colonization and infection^2,15,16^.

As reported in this but also in other studies achieving a proven diagnosis of IPA in non neutropenic patients is challenging. Clinical manifestations such as fever or cough are non specific, and the conventional radiologic features of halo sign and air crescent sign are present in only a minority of non neutropenic patients^2^.

Based on this underlying conditions in the critically ill other diagnostic tools such as the non culture based assay detection of Glactomannan (GM) play a crucial role^17,18^.

Typically angioinavsive growth of *Aspergillus spp*. is more commonly found in neutropenic patients as compared to non-neutropenic patients^19^. This might be the reason that serum GM detection is more reliable in neutropenic patients^19^. Because of the high false negative results in the lack of neutropenia the ESCMID –ECMM-ERS guideline of 2017 graded the use of GM in blood samples in ICU patients only with C, however, using the GM from bronchoalveolar lavage (BAL) it is graded with A and have been proven to be more advantageous in the non neutropenic population^17^.

Repeated serum GM testing was used in this study in only four patients to rule out IPA. All had CHILD B liver cirrhosis, short ICU stay (mean 3 days) and non of them were mechanical ventiltaed, therefore, in combination of clinical and radiological signs IPA could be ruled out.

Altough widley tested in hematological patients only small experiences are available in non-neutropenic patients in using the BAL GM for screening or monitoring of IPA^20,21^.

At the index cutoff value of 0.5–1 ODI, the test yielded a sensitivity up to 100% and specifity ranging from 75% to 92%^20^.

In our study BAL GM testing was not only used for screening in critically ill patients with liver cirrhosis for the first time but also the performance of BAL GM in our study was consistent with previous studies^20,22^.

These findings present that screening for IPA with BAL GM is useful to diagnose IPA in critically ill patients with end stage liver cirrhosis foremost if clinical and radiological signs are not specific and this is in the line with the latest guideline recommendations^17,18^

Standard cultures are still relevant as presented in our study were a growth of *Aspergillus spp*. in 80% on microbiological plates could be observed. These results may not only be interesting for species identification and epidemiological purpose but also in resistance testing if necessary^17,18^.

Other diagnostic tools such as the 1,3-beta-D-Glucan, PCR or bed side tests like the lateral flow device are not yet universally standardized and therefore need further investigation in non-neutropenic patients, but may be promising in detecting or excluding IPA in critically ill patients in the near future^23^.

Not only early diagnosis but also starting therapy at the stage of possible infection, as opposed to proven or probabale infection, might improve survival rates^2^.

Despite the many therapeutic options available today, the mortality rate of IPA in non-neutropenic patients is still high as 90% or like in our cohort 100%. However, in contrast to neutropenic patients, no consensus exists about the exact timepoint for starting empirical therapy at high risk for IPA without evidence for IPA^2^. This might explain the higher rates of IPA in non-neutropenic patients. However, referring to our data, in patients with a high APACHE II, SOFA as well MELD score and an expected longer ICU stay an antimycotic prophylaxis should be discussed.

In our cohort all patients in the IPA group receievd antimycotic therapy. One patient received voriconazole and eleven patients received liposomal amphotericin B. Even though GM testing during mould active therapy is not recommended the decreasing of the ODI in follow up examinations after therapy initiation presents sufficient therapy activity.

To date, the antifungal agents licensed for the first line treatment of IPA include voriconazole, isavuconazole and amphotericin B and its lipid formulations^17,18^. However, as also stated in the latest ESCMID-ECMM-ERS guidelines liposomal amophtericin B is usually the first therapeutic option, especially in patients with liver insufficiency^17,24,25^.

Using liposomal amphotericin B (3 mg per Kg BW) in eleven out of tweleve patients in this study was based on the reason that voriconazole is dialyzible during renal replacement therapy^17,26^. Application of voriconazole oraly by a feeding tube to receive a higher bioavailibilty was due to preexisting esophageal varizes not possible.

The role of new antimycotics in liver insufficiency e.g. isavuconazole have to be evaluted in future studies as promising datas in patients with neutropenia or allo-HSCT are still available.

To our knowledge this is the largest study in critically ill patients with endstage liver cirrhosis evaluating the prevalence and outcome of IPA and the usefulness of repeated GM BAL screening testing in this cohort.

Although, we see the strenght of our results our study has several limitations: First, the study is conducted as a single center study, second the study has an observational character without interventions and third we used modified EORTC/MSG criteria defining IPA.

## Methods

The study was conducted at the medical ICU of the University Hospital Technische Universität München, Germany. We analyzed the medical records of all critically ill patients with liver cirrhosis that were treated at the ICU between April 2016 and April 2018.

All critically ill patients with underlying liver cirrhosis (18 years of age or older) transfered to the ICU were considered eligible for study inclusion. Patients were only included once in the study.

Liver cirrhosis and decompensation were diagnosed either by histological specimen, by ultrasound and/or computed tomography and by clinical criterias for instance ascites or esophageal varices.

This observational study without any specific intervention was reviewed and approved by the ethics committee of our university hospital Klinikum rechts der Isar (5535/12), Technische Universität München, Germany, and all data were processed anonymously. All research was performed in accordance with relevant guidelines/regulations. Written informed consent was obtained by the patient or their legal representatives.

### Diagnostic methods

As a standrad procedure at our ICU all patients with respiratory failure (including cough, dyspnea, hemoptysis) or after orotracheal intubation receive bronchoalveolar lavage (BAL) and computed tomography (CT) of the chest. Both procedures were performed initially after admission to the ICU.

Beside standard microbiological and virological testing, GM from BAL was routinely tested. BAL (including microbiological, virological and GM testing) was weekly performed in patients with mechanical ventilation or in case of worsening of the respiratory situation.

From all not mechanical ventilated patients a serum sample for GM was collected initially after ICU admission and then twice weekly until patients were discharged from the ICU. CT-scans of the chest in these patients were adapted to the clinical presentation and/or positive serum GM testing.

GM antigen in serum and BAL fluid was detected using a sandwich immunocapture ELISA (Platelia Aspergillus; Bio-Rad, Marnes-la-Coquette, France), according to the manufacturer’s recommendations. Positive and negative controls were included in each assay. A result was considered positive in duplicate tests with an optical density index (ODI) values of >0.5 for serum and >1 for BAL samples.

Fungals were detected by direct examination and culture. When fungi are specifically suspected, additional procedures include a direct examination using calcofluor (Becton Dickinson) and culture onto Sabouraud chloramphenicol horse blood agar (Biotrading) were used.

CT scans were performed in all patients after orotracheal intubation or depending on the clinical respiratory situation (cough, dyspnea, pleuritic pain, hemoptysis).

### Diagnostic criteria

IPA was classified according to the slightly modified criterias proposed by the European Organization for Research and Treatment of Cancer/Invasive Fungal Infections Cooperative Group and the National Institute of Allergy and Infectious Diseases Mycoses Study Group (EORTC/MSG) Consensus Group (Table 3)^13^.

**Table 3:** Modified Diagnostic criteria.

Diagnostic criteria
**Proven IPA**
Histopathologic, cytopathologic or direct microscopic examination of tissue invasion by septated, acutely branching filamentous fungi and/or by positive culture for *Aspergillus* in normally sterile specimen
**Probable IPA**
*Host risk factor*: ***Liver cirrhosis***
**AND**
*Clinical radiological features*: abnormal radiological imaging compatible with lung infection
**AND**
*Mycological evidence*: direct test from BAL (cytology, direct microscopy or cultures) indicating the presence of *Aspergillus spp*. or galactomannan antigen detected in serum (ODI > 0, 5) or BAL (ODI > 1)
**Possible IPA**
Presence of a host factor and classical radiological features (dense, well-circumscribed lesion(s) with or without halo sign, air crescent sign and cavity), but absence of mycological criteria

Invasive pulmonary aspergillosis (IPA).

The guidelines were initially designed for patients with underlying hematological malignancies, however, since EORTC inition in 2008, modified criterias were used in several studies evaluating prevalence of IPA in non neutropenic patients. Modifications for this study were necessary because host factors in these guidelines were originally defined for patients with underlying hematological malignancies. Liver cirrhosis is not included. Therefore, liver cirrhosis was added as a host risk factor.

### Statistical analysis

We used IBM SPSS Statistics 23 (SPSS inc., Chicago, IL, USA) for all statistical analyses in this study. To present descriptive statistics, we calculated mean ± standard deviation for normally distributed continuous data, and absolute and relative frequencies for categorical

Data (Fishers exact test). To compare the other variables, we performed the t-test for paired samples and the Wilcoxon signed rank test for paired samples for normally distributed data and not normally distributed data, respectively. A p-value below a significance level of 5% (p < 0.05) indicates statistical significance.

Multivariate logistic regression analysis was performed by backward selection procedures to identify predictors of death using variables identified univariate analysis (p < 0.10).

The diagnostic performance of GM test was evaluated as sensitivity and specificity, calculated using the mean parameters, with 95% confidence intervals (CI).

### Ethics approval and consent to participate

This observational study without any specific intervention was reviewed and approved by the ethics committee of our university hospital Klinikum rechts der Isar (5535/12), Technische Universität München, Germany, and all data were processed anonymously. Written informed consent was obtained by the patient or their legal representatives.

## Conclusion

Invasive pulmonary aspergillosis is not a rare entity in critically ill patients with liver cirrhosis.

As presented in our study screening for IPA in patients with end stage liver cirrhosis using the GM from BAL is indicated, easy to perform and a necessity to improve outcome of critically ill patients with endstage liver cirrhosis.

IPA has a relevant impact on outcome of patients with end stage liver disease. This is not only expressed in higher APACHE II, SOFA and MELD score and longer ICU stay but also in a devasting high mortality rate as compared to No-IPA patients.

Implementation of prophylactic antimycotic therapy strategies have to be discussed in this special group of patients and further studies have to be performed.

## Declarations

### Ethics approval and consent to participate

This observational study without any specific intervention was reviewed and approved by the ethics committee of our university hospital Klinikum rechts der Isar (5535/12), Technische Universität München, Germany, and all data were processed anonymously. Written informed consent was obtained by the patient or their legal representatives.

### Consent for publication

Not applicable.

## Data Availability

The authors confirm that, for approved reasons, some access restrictions apply to the data underlying the findings. Due to ethical and legal restrictions, confidential data are available upon request. To receive anonymized data readers are welcome to contact the corresponding author.

## References

[1] Steinbach William J., Marr Kieren A., Anaissie Elias J., Azie Nkechi, Quan Shun-Ping, Meier-Kriesche Herwig-Ulf, Apewokin Senu, Horn David L. (2012). Clinical epidemiology of 960 patients with invasive aspergillosis from the PATH Alliance registry. Journal of Infection.

[2] Bassetti, M., Peghin, M. & Vena, A. Challenges and Solution of Invasive Aspergillosis in Non-neutropenic Patients: A Review. *Infect Dis Ther*. Mar; **7**(1), 17–27 (2018). 10.1007/s40121-017-0183-9 PMC5840102 29273978

[3] Lahmer Tobias, Messer Marlena, Mayr Ulrich, Saugel Bernd, Noe Sebastian, Schultheiss Caroline, Thies Philipp, Spinner Christoph, Nennstiel Simon, Schwerdtfeger Christiane, Phillip Veit, Schmid Roland M., Huber Wolfgang (2014). Fungal “colonisation” is Associated with Increased Mortality in Medical Intensive Care Unit Patients with Liver Cirrhosis. Mycopathologia.

[4] Jeurissen S, Vogelaers D, Sermijn E, Van Dycke K, Geerts A, Van Vlierberghe H, Colle I (2013). INVASIVE ASPERGILLOSIS IN PATIENTS WITH CIRRHOSIS, A CASE REPORT AND REVIEW OF THE LAST 10 YEARS. Acta Clinica Belgica.

[5] Vaezi A. *et al*. Frequency and Geographic Distribution of CARD9 Mutations in Patients With Severe Fungal Infections. *Front Microbiol*. Oct 12; **9**, 2434 (2018). 10.3389/fmicb.2018.02434 PMC6195074 30369919

[6] Albillos Agustín, Lario Margaret, Álvarez-Mon Melchor (2014). Cirrhosis-associated immune dysfunction: Distinctive features and clinical relevance. Journal of Hepatology.

[7] Gomez, F., Ruiz, P. & Schreiber, A. D. Impaired function of macrophage Fc gamma receptors and bacterial infection in alcoholic cirrhosis. *N Engl J Med*. Oct 27; **331**(17), 1122–8 (1994). 10.1056/NEJM199410273311704 7935636

[8] Fiuza C, Salcedo M, Clemente G, Tellado JM (2000). *In vivo* neutrophil dysfunction in cirrhotic patients with advanced liver disease. J Infect Dis. Aug.

[9] Bonnel, A. R., Bunchorntavakul, C. & Reddy, K. R. Immune dysfunction and infections in patients with cirrhosis. *Clin Gastroenterol Hepatol*. Sep; **9**(9), 727–38 (2011). 10.1016/j.cgh.2011.02.031 21397731

[10] Coant Nicolas, Simon-Rudler Marika, Gustot Thierry, Fasseu Magali, Gandoura Sonia, Ragot Kévin, Abdel-Razek Waël, Thabut Dominique, Lettéron Philippe, Ogier-Denis Eric, Ouziel Romy, Devière Jacques, Lizard Gérard, Tellier Zéra, Lebrec Didier, Moreau Richard (2011). Glycogen synthase kinase 3 involvement in the excessive proinflammatory response to LPS in patients with decompensated cirrhosis. Journal of Hepatology.

[11] Lahmer Tobias, Messer Marlena, Schwerdtfeger Christiane, Rasch Sebastian, Lee Marcel, Saugel Bernd, Schmid Roland M., Huber Wolfgang (2014). Invasive Mycosis in Medical Intensive Care Unit Patients with Severe Alcoholic Hepatitis. Mycopathologia.

[12] Karakike E, Moreno C, Gustot T (2017). Infections in severe alcoholic hepatitis. Ann Gastroenterol.

[13] De Pauw, B. *et al*. European Organization for Research and Treatment of Cancer/Invasive Fungal Infections Cooperative Group; National Institute of Allergy and Infectious Diseases Mycoses Study Group (EORTC/MSG) Consensus Group. Revised definitions of invasive fungal disease from the European Organization for Research and Treatment of Cancer/Invasive Fungal Infections Cooperative Group and the National Institute of Allergy and Infectious Diseases Mycoses Study Group (EORTC/MSG) Consensus Group. *Clin Infect Dis*. Jun 15; **46**(12), 1813–21 (2008). 10.1086/588660 PMC2671227 18462102

[14] Prattes Juergen, Hoenigl Martin, Krause Robert, Buzina Walter, Valentin Thomas, Reischies Frederike, Koidl Christoph, Zollner-Schwetz Ines (2017). Invasive aspergillosis in patients with underlying liver cirrhosis: a prospective cohort study. Medical Mycology.

[15] Meersseman W (2007). Invasive aspergillosis in the intensive care unit. Clin Infect Dis..

[16] Singer M, Deutschman CS, Seymour CW (2016). The Third International Consensus Definitions for Sepsis and Septic Shock (Sepsis-3). JAMA.

[17] Ullmann, A. J. *et al*. Diagnosis and management of Aspergillus diseases: executive summary of the 2017 ESCMID-ECMM-ERS guideline. *Clin Microbiol Infect* May; **24** (Suppl 1), e1–e38 (2018). 10.1016/j.cmi.2018.01.002 29544767

[18] Patterson, T. F. *et al*. Practice Guidelines for the Diagnosis and Management of Aspergillosis: 2016 Update by the Infectious Diseases Society of America. *Clin Infect Dis* Aug 15; **63**(**4**), e1–e60 (2016). 10.1093/cid/ciw326 PMC4967602 27365388

[19] Hope WW, Walsh TJ, Denning DW (2005). Laboratory diagnosis of invasive aspergillosis. Lancet Infect Dis.

[20] Meersseman W (2008). Galactomannan in bronchoalveolar lavage fluid: a tool for diagnosing aspergillosis in intensive care unit patients. Am J Respir Crit Care Med.

[21] Guinea J (2008). Value of a single galactomannan determination (platelia) for the diagnosis of invasive aspergillosis in non-hematological patients with clinical isolation of Aspergillus spp. Med Mycol.

[22] Zou M (2012). Systematic review and meta-analysis of detecting galactomannan in bronchoalveolar lavage fluid for diagnosing invasive aspergillosis. PLoS One.

[23] Hoenigl M., Prattes J., Spiess B., Wagner J., Prueller F., Raggam R. B., Posch V., Duettmann W., Hoenigl K., Wolfler A., Koidl C., Buzina W., Reinwald M., Thornton C. R., Krause R., Buchheidt D. (2014). Performance of Galactomannan, Beta-D-Glucan, Aspergillus Lateral-Flow Device, Conventional Culture, and PCR Tests with Bronchoalveolar Lavage Fluid for Diagnosis of Invasive Pulmonary Aspergillosis. Journal of Clinical Microbiology.

[24] Tissot Frederic, Agrawal Samir, Pagano Livio, Petrikkos Georgios, Groll Andreas H., Skiada Anna, Lass-Flörl Cornelia, Calandra Thierry, Viscoli Claudio, Herbrecht Raoul (2016). ECIL-6 guidelines for the treatment of invasive candidiasis, aspergillosis and mucormycosis in leukemia and hematopoietic stem cell transplant patients. Haematologica.

[25] Linden PK, Coley K, Fontes P, Fung JJ, Kusne S (2003). Invasive aspergillosis in liver transplant recipients: outcome comparison of therapy with amphotericin B lipid complex and a historical cohort treated with conventional amphotericin B. Clin Infect Dis.

[26] Falcone, M., Massetti, A. P., Russo, A., Vullo, V. & Venditti, M. Invasive aspergillosis in patients with liver disease. *Med Mycol* May; **49**(4), 406–13 (2011). 10.3109/13693786.2010.535030 21108575

